# Supplementary material for: Characterization of the complete mitochondrial genome of Orthaga olivacea Warre (Lepidoptera Pyralidae) and comparison with other Lepidopteran insects
Source: PLoS One. 2020 Mar 6;15(3):e0227831. doi: 10.1371/journal.pone.0227831 (PMC7059908; doi:10.1371/journal.pone.0227831)
Supplement: Attachment — Submitted filename: Response to Reviewers.docx [file pone.0227831.s001.docx]

Dear Editors and Reviewers:

Thank you very much for your letter and for the reviewers’ comments concerning our manuscript entitled “Characterization of the complete mitochondrial genome of *Orthaga olivacea* Warre (Lepidoptera Pyralidae) and comparison with other Lepidopteran insects” (ID: PONE-D-19-24590). Those comments are all valuable and very helpful for revising and improving our paper, as well as the important guiding significance to our researches. We have studied comments carefully and have made correction which we hope meet with approval. Revised portion are marked in color in the paper. The main corrections in the paper and the responds to the reviewer’s comments are as flowing:

Responds to the reviewer’s comments:

Reviewer #1:

Major issues:

1. Response to comment: (Pyraloidea taxon sampling. Line 62. “Considering the limited information of the mitochondrial sequences in Pyralidae, we sequenced the complete mitogenome of Orthaga olivacea, and compared it with other insect species, especially with the members of Pyralidae species.” This is a reasonable justification for sequencing the mitogenome of O. olivacea, but it is very curious that only 3 mitogenomes were included in the phylogenetic analysis and many of the other pyralid mitochondrial genomes that are available from Genbank were not included in these analyses including Corcyra cephalonica, Amyelois transitella, Plodia interpunctella (3 mitogenomes), Ephestia kuehniella (3 mitogenomes), Meroptera pravella, and Hypsopygia regina. Similarly, the sister-family to the Pyralidae includes an even larger number of species with sequenced mitochondrial genomes that were not included in the presented analyses including Paracymoriza prodigalis, Elophila interruptalis, Pseudargyria interruptella, Chilo auriculius, Chilo sacchariphagus, Evergestis junctalis, Nomophila noctuella, , Tryspandoes striata, Glyphodes quadrimaculalis, Spoladea recurvalis, Dichocrocis punctiferalis, Glyphodes pyloalis, Maruca vitrata, Maruca testulalis, Haritalodes derogate, Pycnarmon lactiferalis, Loxostege stricticalis, Endotricha consoci, Euzophera pyriella, Dichocrocis punctiferalis, and Cnaphalocrocis medinalis (3 mitogenomes). If one of the goals of the authors is to demonstrate that Orthaga olivacea belongs within the Pyralidae and to determine its closest relatives with sequences mitochondrial genomes, then they need to repeat their phylogenetic analysis after supplementing their current data set with all of these additional species. (Also note that Lista haraldusalis is misspelled in Fig. 7 and in other locations in the manuscript. Also, Family Pyralidae (and probably also Family Crambidae) should be indicated in Fig. 7.))

Response: We are very sorry for our omission that it is inadequate to the goals to demonstrate that Orthaga olivacea belongs within the Pyralidae and to determine its closest relatives with sequences mitochondrial genomes with only 3 pyralid mitochondrial genomes were included in the phylogenetic analysis. According reviewer’s suggestion, we have repeated our phylogenetic analysis after supplementing our current data set with all of these additional species. And we have corrected the misspelled of *Lista haraldusalis* in Fig. 7 and in other locations in the manuscript. Also, Family Pyralidae (and probably also Family Crambidae) was indicated in Fig. 7.

2. Response to comment: (The authors employ 2 non-Lepidoptera outgroup species: Drosophila yakuba, a fly (Order Diptera) and Locusta migratoria, a grasshopper (Order Orthoptera), but the authors do not include any representatives of the insect Order most closely related to the Lepidoptera, the caddisflies (Order Trichoptera). There are at least 17 complete mitochondrial genomes representing several caddisfly Families available through Genbank (Al-Baeity et al. 2019). To root the Lepidopteran tree properly caddisflies sequences MUST also be included in the phylogenetic analyses.)

Response: Considering the Reviewer’s suggestion, we have included the caddisflies sequences of Limnephilus hyalinus in the phylogenetic analyses as outgroup.

Minor issues:

1. Response to comment: (Line 2. Title: In modern usage, the lepidopteran family is usually called Pyralidae, not “Pyralididae”. Change here and throughout manuscript.)

Response: We are very sorry for our Negligence of the use of “Pyralididae”, and we have corrected it to “Pyralidae” throughout manuscript.

2. Response to comment: (Line 18. Abstract: Suggested reword with greater specificity “Orthaga olivacea Ware (Lepidoptera Pyralidae) is an important agricultural pest of camphor trees (Cinnamomum camphora).”)

Response: According reviewer’s suggestion, we have reworded with greater specificity.

3. Response to comment: (Line 19. Suggested reword “To further supplement the known genome-level…”)

Response: According reviewer’s suggestion, we have reworded it in the target location.

4. Response to comment: (Line 20. Suggested reword “…other species of Lepidoptera.”)

Response: According reviewer’s suggestion, we have reworded it in the target location.

5. Response to comment: (Lines 31-31. Suggested reword “Phylogenetic analysis suggested that among sequenced lepidopteran mitochondrial genomes, Orthaga olivacea Warre was most closely related…”)

Response: According reviewer’s suggestion, we have reworded it in the target location.

6. Response to comment: (Line 38. Suggested reword “…(mtDNA) is a circular molecule range in size from 14 to 19 kb…”)

Response: According reviewer’s suggestion, we have reworded it in the target location.

7. Response to comment: (Line 42. Suggested reword “…A+T-rich region, the largest noncoding…”)

Response: According reviewer’s suggestion, we have reworded it in the target location.

8. Response to comment: (Line 43-47. Suggested reword to remove repetition “Whole mitochondrial genomes are a useful data source for several research areas, such as evolutionary genomics (9, 10), comparative molecular evolution (11, 12), phylogeography (13), and population genetics (14).)

Response: According reviewer’s suggestion, we have removed repetition in the target location.

9. Response to comment: (Line 50-51. Suggested reword to remove extraneous information “…over 25,000 species and some pyralids are important agricultural pests…”)

Response: According reviewer’s suggestion, we have removed extraneous information in the target location.

10. Response to comment: (Line 53-53. Suggested reword “Despite their diversity and great importance as pests of agricultural and forestry plants, they are also valuable for pollinating plants of economic importance. Most species in the family Pyralidae do not yet have sequenced mitogenomes.”)

Response: According reviewer’s suggestion, we have reworded it in the target location.

11. Response to comment: (Line 58. “remove” should be “removal”)

Response: According reviewer’s suggestion, we have corrected “remove” to “removal”.

12. Response to comment: (Line 59-60. “However, overlapping generations and irregularity of abundance in the field from May to October make it very difficult to control.”)

Response: According reviewer’s suggestion, we have reworded it in the target location.

13. Response to comment: (Line 72. Suggested reword “…the camphor trees on the campus of…”)

Response: According reviewer’s suggestion, we have reworded it in the target location.

14. Response to comment: (Line 94. “…insert DNA were sequenced at least three times…” Query: was sequencing of the inserts done in both directions? If yes, please specify in the text.)

Response: Yes, the sequencing of the inserts was done in both directins. According reviewer’s suggestion, we have specified in the text.

15. Response to comment: (Lines 99-100. Suggested reword “…under the accession number MN078362.”)

Response: According reviewer’s suggestion, we have reworded it in the target location.

16. Response to comment: (Line 125-126. “…mitogenome sizes documented for other sequenced lepidopterans which range from 14,534 bp in Ostrinia nublilalis (incomplete)…” Since the sequencing of the mitochondrial genome of O. nublilalis is incomplete, it is inappropriate and incorrect to use this sequence to estimate the minimum mitochondrial genome size in the Lepidoptera. This data point should be replaced with the smallest completely sequenced mitochondrial genome from the Lepidoptera.)

Response: Thank you for pointing out the error. We have re-searched NCBI, and found that the sequencing of the mitochondrial genome of *Ostrinia nublilalis* is complete with 14,535 bp. And maybe *Ostrinia nublilalis* is the smallest completely sequenced mitochondrial genome from the Lepidoptera. we have corrected it in the target location.

17. Response to comment: (Lines 136-137. Suggested reword “In addition, the GC skew…”)

Response: According reviewer’s suggestion, we have reworded it in the target location.

18. Response to comment: (Line 146. Table 4. I’m not sure that this table is necessary and perhaps should be removed.)

Response: Thank you for your suggestion, but we think by base preference and compared it with other species in table 4, can better understand this mitochondrial genome. Therefore, we chose to keep table 4 in the manuscript.

19. Response to comment: (Line 166. Suggested reword “…observed in most other lepidopteran mitogenomes and are…”)

Response: According reviewer’s suggestion, we have reworded it in the target location.

20. Response to comment: (Line 177-179. Suggested reword “…for instance, L. haraldusalis, G. mellonella, B. mori, B. thibetaria, and L. malifoliella species all lack GCT codons, while G. mollesta lacks CGT codons.”)

Response: According reviewer’s suggestion, we have reworded it in the target location.

21. Response to comment: (Line 246-248. “The species Orthaga olivacea…” This sentence should be revised based on the updated phylogenetic analysis after adding the taxa I suggested in the major revisions section above.)

Response: According reviewer’s suggestion, we have revised this sentence based on the updated phylogenetic analysis.

22. Response to comment: (Lines 252-253. Suggested reword “…constituent with prior studies of lepidopteran phylogeny.”)

Response: According reviewer’s suggestion, we have reworded it in the target location.

Reviewer #2:

1. Response to comment: (the tables would be “three line”.)

Response: According reviewer’s suggestion, we have changed all tables into “three line” forms.

2. Response to comment: (the literature 34 (line 353) was not complete.)

Response: According reviewer’s suggestion, we have reworded it in the target location.

3. Response to comment: (“Warre” (ects.) in the figures would not be italic.)

Response: We are very sorry for our error application of the italic of “Warre” (ects.) in the figures, we have corrected it in all figures.

4. Response to comment: (“correct others, for examples, line 194 “TRNAs” (tRNAs ?); line 201 “The rNAs” (The rRNAs ?), ect.)

Response: We are very sorry for our incorrect in spelling, and we have already corrected them in the text and marked in color.

Reviewer #3:

1. Response to comment: (Introduction: the authors should provide clearly the study aim and scientific questions. It includes a description of the importance of the research and the study and reviews most of the previous literature. However, the authors have omitted a few studies of relevance and these should be included,)

Response: According reviewer’s suggestion, we have reworded the Introduction section to provide a clearer outline of the study aims and research question and marked in color.

2. Response to comment: (It is not clear from the manuscript that the collected Orthaga olivacea samples were verified as truly belongs to the said species. It is suggested for the author to delimit the detailed morphological characters of the species to confirm.)

Response: According reviewer’s suggestion, we have added detailed description of the morphological characteristics of the species' larvae in the materials and methods section to better delimit the species.

3. Response to comment: (Based on the dataset of 13 concatenated protein sequences, the authors reconstructed the phylogeny of Lepidoptera using MEGA with the Maximum Likelihood method. It is more persuasive and popular to carry out such analysis with RAxML method.)

Response: We are very sorry that we didn’t reconstruct the phylogeny of Lepidoptera using the RAxML method as you suggested. Because we think the RAxML method is an alternative solution in phylogeny, we found that using MEGA method to analyze the phylogeny of Lepidoptera is also popular. For example, in the study of *Cerura menciana* (*Dai et. al., 2015*), *Biston marginata* (*Zheng et al., 2018*) and *Ctenoptilum vasava* (*Hao et. al., 2012*) in Lepidoptera, they used MEGA to reconstruct the evolutionary relationship of Lepidoptera with the Maximum Likelihood method, and also got a better evolutionary relationship tree. In this study, based on the analysis of the original evolutionary relationship, we added another 25 species of Pyralidae and finally got a better evolutionary relationship of Lepidoptera. So we think that the MEGA method can also be used to construct the evolutionary tree based on Lepidoptera mitochondria.

4. Response to comment: (There are some errors in grammar and syntax throughout the text of the manuscript, the English writing should be further improved.)

Response: We are very sorry for our incorrect in grammar and syntax, and we have already corrected them in the text and marked in color.

We tried our best to improve the manuscript and made some changes in the manuscript. These changes will not influence the content and framework of the paper. And here we did not list the changes but marked in revised manuscript. We appreciate for Editors/Reviewers’ warm work earnestly, and hope that the corrections will meet with approval. Once again, thank you very much for your comments and suggestions.

Yours Sincerely

Guoqing Wei
