# Supplementary material for: Characterization of the complete mitochondrial genome of Orthaga olivacea Warre (Lepidoptera Pyralidae) and comparison with other Lepidopteran insects
Source: PLoS One. 2020 Mar 6;15(3):e0227831. doi: 10.1371/journal.pone.0227831 (PMC7059908; doi:10.1371/journal.pone.0227831)
Supplement: Attachment — Submitted filename: Response to Reviewers.docx [file pone.0227831.s002.docx]

Dear Editor:

Thank you very much for your letter and the comments concerning our manuscript entitled “Characterization of the complete mitochondrial genome of *Orthaga olivacea* Warre (Lepidoptera Pyralidae) and comparison with other Lepidopteran insects” (ID: PONE-D-19-24590). Those comments are all valuable and very helpful for revising our paper to meet the acceptable criterion for publication in PLOS ONE. We have studied comments carefully and have made corrections which we hope to meet with approval. Revised portions are marked in color in the manuscript. The main corrections in the paper and the responds to the comments are as flowing:

Responds to the reviewer’s comments:

1. Response to comment: (Line 62. Delete entire sentence beginning with "What'more considering the limited..." It is unnecessary.)

Response: According to the reviewer’s suggestion, we have deleted it in the target location.

2. Response to comment: (Table 2 includes a column of references. These table citations are not in the same format as the in-text citations in the rest of the manuscript and some of these references do not appear in the reference section at the end of the manuscript. Please correct the formatting, and ensure that all of the references listed in Table 2 also appear in the reference section.)

Response: We are very sorry for our negligence of the citations format and omission of some references in Table 2, and we have corrected the formatting in Table 2 and increased the omissive references in the reference section.

3. Response to comment: (The reference for the Meroptera pravella mitochondrial genome in Table 2 is listed as "Consortium et al. (2017)". This is properly referenced as "Living Prairie Consortium (2017)".)

Response: Considering the second suggestion, we have modified the reference formats in table 2.

4. Response to comment: (Fig. 7. The vertical line associated with the label "Pyraloidea" should extend from Glyphodes quadrimaculalis to Ephestia kuehniella in this figure.)

Response: According the suggestion, we modified the vertical line associated with the label "Pyraloidea" and make sure it is extended from *Glyphodes quadrimaculalis* to *Ephestia kuehniella* in fig. 7.

We tried our best to improve the manuscript and made some changes in the manuscript. These changes will not influence the content and framework of the paper. And here we did not list the changes but marked in revised manuscript. We appreciate for Editors/Reviewers’ warm work earnestly, and hope that the corrections will meet with approval. Once again, thank you very much for your comments and suggestions.

Yours Sincerely

Guoqing Wei
